# EMBL2checklists: A Python package to facilitate the user-friendly submission of plant and fungal DNA barcoding sequences to ENA

**DOI:** 10.1371/journal.pone.0210347

**Published:** 2019-01-10

**Authors:** Michael Gruenstaeudl, Yannick Hartmaring

**Affiliations:** 1 Institut für Biologie, Freie Universität Berlin, Berlin, Germany; 2 Institut für Bioinformatik, Freie Universität Berlin, Berlin, Germany; University of Helsinki, FINLAND

## Abstract

**Background:**

The submission of DNA sequences to public sequence databases is an essential, but insufficiently automated step in the process of generating and disseminating novel DNA sequence data. Despite the centrality of database submissions to biological research, the range of available software tools that facilitate the preparation of sequence data for database submissions is low, especially for sequences generated via plant and fungal DNA barcoding. Current submission procedures can be complex and prohibitively time expensive for any but a small number of input sequences. A user-friendly software tool is needed that streamlines the file preparation for database submissions of DNA sequences that are commonly generated in plant and fungal DNA barcoding.

**Methods:**

A Python package was developed that converts DNA sequences from the common EMBL and GenBank flat file formats to submission-ready, tab-delimited spreadsheets (so-called ‘checklists’) for a subsequent upload to the annotated sequence section of the European Nucleotide Archive (ENA). The software tool, titled ‘EMBL2checklists’, automatically converts DNA sequences, their annotation features, and associated metadata into the idiosyncratic format of marker-specific ENA checklists and, thus, generates files that can be uploaded via the interactive Webin submission system of ENA.

**Results:**

EMBL2checklists provides a simple, platform-independent tool that automates the conversion of common DNA barcoding sequences into easily editable spreadsheets that require no further processing but their upload to ENA via the interactive Webin submission system. The software is equipped with an intuitive graphical as well as an efficient command-line interface for its operation. The utility of the software is illustrated by its application in four recent investigations, including plant phylogenetic and fungal metagenomic studies.

**Discussion:**

EMBL2checklists bridges the gap between common software suites for DNA sequence assembly and annotation and the interactive data submission process of ENA. It represents an easy-to-use solution for plant and fungal biologists without bioinformatics expertise to generate submission-ready checklists from common DNA sequence data. It allows the post-processing of checklists as well as work-sharing during the submission process and solves a critical bottleneck in the effort to increase participation in public data sharing.

## Introduction

Only a few software tools assist in the preparation of DNA sequence data for submission to public sequence databases, despite the centrality of this process for disseminating novel biological data. Contemporary biological research depends on the preservation, curation, and reproducibility of the analyzed data [1, 2], and their submission to publicly accessible databases constitutes one of the most important practices in biology [3, 4], particularly in the era of big data [5]. DNA sequences generated to identify and characterize novel organisms or unchartered biodiversity must typically be submitted to public sequence databases before publication of the research is granted [6, 7]. Compliance with this prerequisite remains mixed [8–10]. Several large nucleotide sequence repositories accept DNA sequence submissions, including GenBank [11], the European Nucleotide Archive [12] and the DNA Data Bank of Japan [13]. These repositories coordinate their policies and operations under the umbrella of the International Nucleotide Sequence Database Collaboration (INSDC; [14]), but each database employs custom procedures for sequence upload and data submission. ENA, for example, channels the submission of annotated DNA sequences through the Webin submission framework (https://www.ebi.ac.uk/ena/submit/sra/; [15]), which, in its interactive version, operates with pre-formatted, tab-delimited spreadsheets. These spreadsheets (also called ‘annotation checklists’ or ‘templates’) are filled out by the user and then uploaded for submission. In order to account for different types of annotated DNA sequences (e.g., coding vs. non-coding, nuclear vs. organellar origin), a series of pre-tailored spreadsheets (hereafter ‘checklists’) was developed by ENA, each with its idiosyncratic, tab-delimited fields of information. Users of ENA must choose the correct spreadsheet for their data submission, and different types of DNA sequences must be submitted via separate data uploads. Since June 2017, the submission process through Webin has been automated and now includes automatic validation procedures for annotation features, taxonomic metadata, and sequence integrity [12]. Despite the centrality of data sharing to biological research, the range of user-friendly software tools that assist in data preparation for database submission is perceived as low [3]. Indeed, very few, if any, user-friendly software tools exist that assist in the preparatory steps necessary prior to uploading DNA sequences to public databases (e.g., the assignment of metadata to individual DNA sequences); software tools that assist with, and are specifically customized for, the preparation of common plant and fungal DNA barcoding sequences are entirely missing.

Unlike sequence submissions to GenBank, the preparation of DNA sequence data for submission to ENA is insufficiently facilitated, highlighting the demand for software that converts annotated DNA sequences into submission-ready checklists. Upon DNA sequencing, researchers often utilize user-friendly software suites such as Artemis [16], DnaSP [17], Geneious [18] or PhyDE [19] for the assembly and annotation of DNA sequences. Some of these suites (e.g., Artemis, DnaSP, Geneious) enable the conversion of annotated DNA sequences to file formats that are easily submittable to GenBank, either by producing files in a direct submission format (i.e., the Sequin format [20]) or through the processing with additional tools that convert GenBank-formatted flat files into the Sequin file format (e.g., GB2sequin [21]). Moreover, bioinformatic pipelines exist that channel raw DNA sequences through data processing and export the final sequences to GenBank, even though some of these pipelines were primarily designed for educative purposes (e.g., the blue line of the DNA Subway project, [22]) or are currently inaccessible (e.g., [23]; access attempted in May and September 2018). Equivalent tools for ENA are currently missing: While there are software suites that generate EMBL-formatted flat files (e.g., Artemis, DnaSP, the tool seqret of EMBOSS [24]), and software tools for command-line validation and submission of flat files are being developed [25], they do not produce output files that are suitable for a direct upload via the interactive Webin submission system of ENA. To the best of our knowledge, no conversion tool currently exists that automatically converts annotated DNA sequences and associated metadata from the EMBL flat file format into submission-ready Webin checklists. The Webin submission system is the default gateway for DNA sequence submission to ENA and offers three routes for data upload (https://ena-docs.readthedocs.io/en/latest/): an interactive route, in which checklists are uploaded or generated online; a programmatic route, in which pre-formatted flat files can be submitted to the ENA server [12], and a command-line route, which is presently being implemented [25]. Flat files in EMBL file format are not accepted through the interactive, but only through the programmatic and command-line submission routes. However, using the programmatic or the command-line route requires bioinformatics expertise and is, thus, inaccessible to many users. In absence of a more intuitive solution, many users are compelled to undergo the tedious process of parsing information from EMBL-formatted flat files and copying them into Webin checklists manually. Some users also employ the guided web interface of the Webin submission platform, in which users click their way through an elaborate data entry interface. For any but a small number of input sequences, the use of the guided web interface is prohibitively time-expensive. Thus, there is a strong demand for an easy-to-use, platform-independent software tool that converts DNA sequences in EMBL flat file format including their annotation features and associated metadata into submission-ready checklists for upload via the interactive Webin submission system.

**Fig 1:**
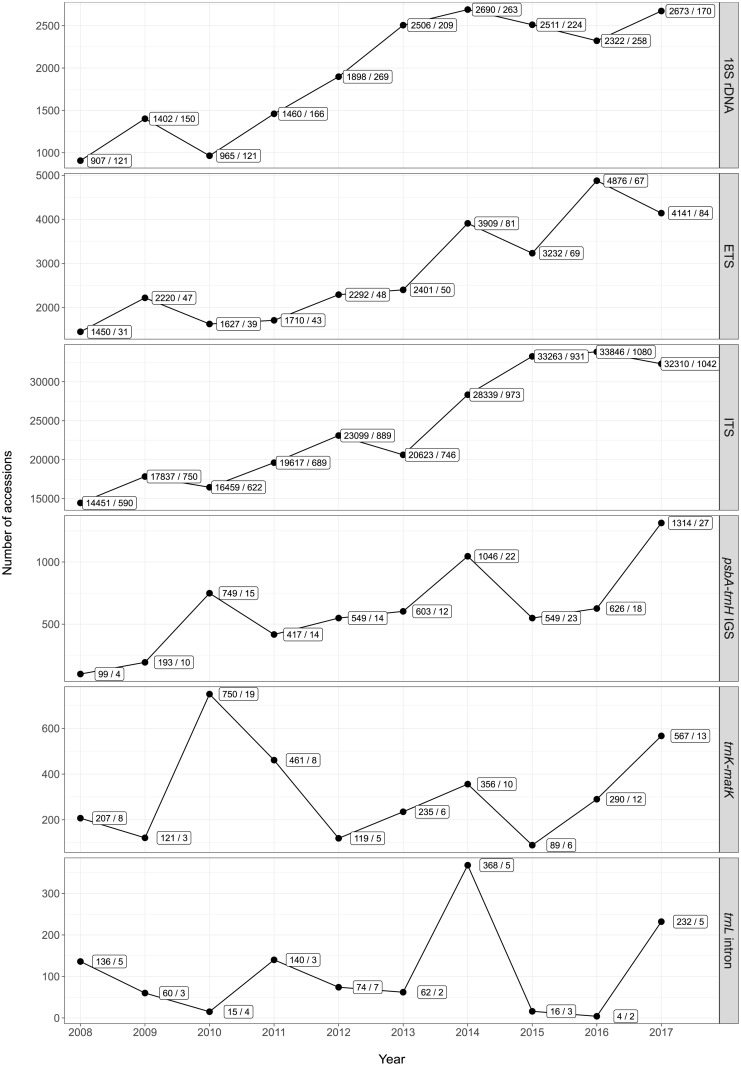
The number of DNA sequences of ENA’s plant taxonomic division (PLN) of the standard annotated assembled sequence data class (STD) released on ENA per calendar year, displayed by type of DNA barcoding marker. Each count of released DNA sequences is followed by the total number of investigations that the sequences are associated with; the two numbers are separated by a forward slash. Due to the daily synchronization of sequence records between GenBank, ENA, and DDBJ, the release numbers are identical across all three databases.

DNA barcoding is a key method to identify and characterize plant and fungal specimens, and tens of thousands of DNA sequences of typical barcoding markers are released by ENA each year. The identification and characterization of plant specimens via the sequencing of specific genome regions (’plant DNA barcodes’; [26]) have become a key method in botanical research [27]. A plethora of DNA sequences have been generated in investigations on suitable plant DNA barcoding markers [28–30], and plant DNA barcoding is now routinely applied across evolutionary, ecological and conservation research [26, 27, 31], even in regional studies [32–34]. A similar situation exists for DNA barcoding in fungi [35, 36]. The number of plant and fungal DNA sequences of typical barcoding regions submitted to, and released by, the large nucleotide sequence repositories GenBank, ENA and DDBJ each year is immense (Fig 1). Over the past decade (2008-2017), a total of 239,844 annotated ITS DNA sequences of ENA’s plant taxonomic division, stemming from no less than 8,312 investigations, have become available via ENA. When summed across six common plant DNA barcoding markers whose submission to ENA is implemented in the software presented here, a total of 297,483 annotated DNA sequences, stemming from no less than 11,110 investigations, were released. Similar numbers exist for annotated DNA sequences of ENA’s fungal taxonomic division (S1 Fig). Due to the regular synchronization of sequence records between GenBank, ENA, and DDBJ, these statistics reflect release numbers across all three databases; current query interfaces do not enable the evaluation which proportion was submitted to ENA directly. If only a fraction of these investigations could be aided through a software tool that streamlines and, thus, expedites their sequence submission, it would make a considerable positive impact on the research community.

DNA sequences generated through DNA barcoding are well suited for a streamlined and automated conversion to marker-specific Webin checklists. Future investigations applying plant or fungal DNA barcoding will invariably require the submission of novel DNA sequences to public sequence repositories [7, 31]. A user-friendly, streamlined data submission process can be instrumental in the data sharing process [3]. DNA barcoding sequences lend themselves for the application of software tools that streamline and automate their submission process, because common barcoding markers display (a) a general homogeneity in sequence length and gene synteny, at least within most target lineages [37, 38], (b) a general absence of structural inversions or strong secondary structure [38, 39], and (c) the presence of conserved regions, preferentially in the flanking parts of the marker, that allow the anchoring of universal PCR primers and, thus, bidirectional sequencing [37–39]. Consequently, DNA sequence data of common barcoding markers are often submitted in large batches per investigation, leading to cases where dozens of sequences are submitted to ENA per investigation. For submissions of annotated ETS DNA sequences, for example, an average of 49 sequences was submitted per investigation over the past decade (Fig 1). In the light of these factors, the preparation process for submissions of DNA sequences that are commonly generated in plant and fungal DNA barcoding experiments is currently insufficiently automated, especially to the annotated sequence section of ENA. A software tool is needed that streamlines and automates the preparation of such barcoding sequences, primarily for submissions via the interactive Webin submission system.

**Fig 2:**
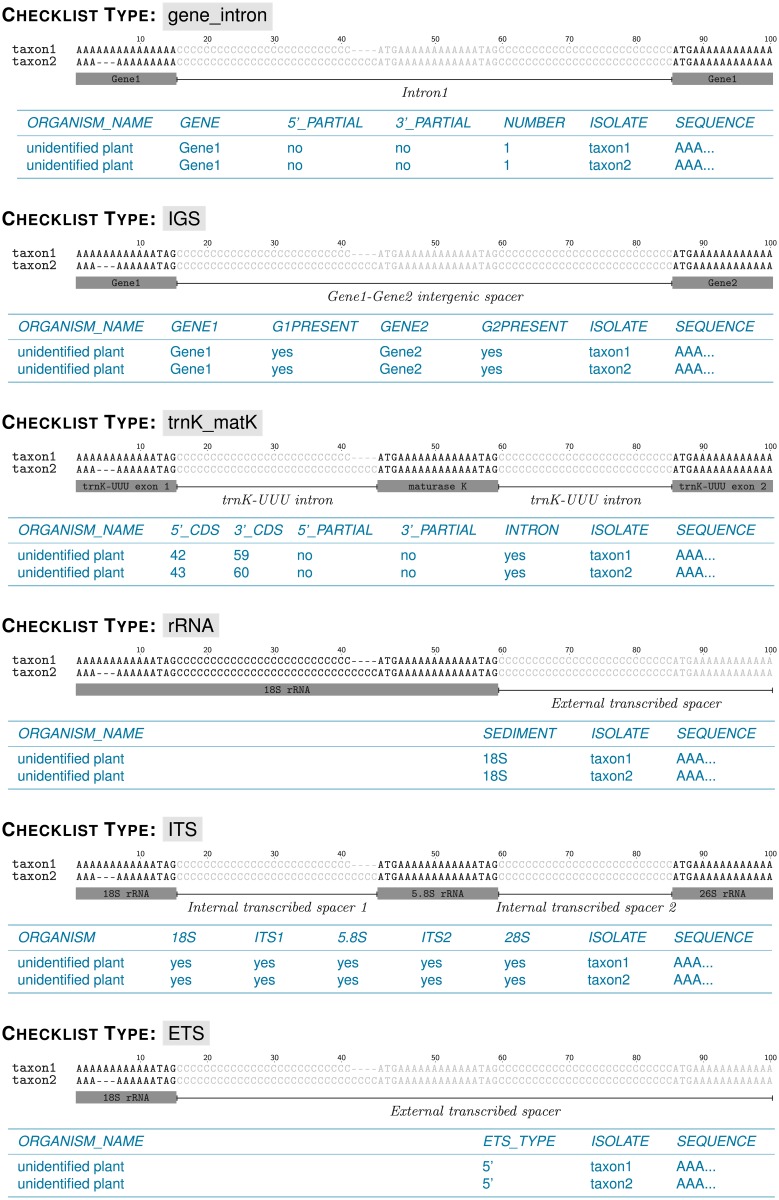
Structural overview of the six DNA markers and their corresponding Webin checklists as implemented in EMBL2checklists. The overview displays the structural characteristics of each DNA marker (in black) and the idiosyncratic column names and column values (in blue) of the corresponding checklists. The column names and column values of each checklist are also presented in Table 2, where they are cross-referenced with the mandatory annotation features and feature qualifiers of the input flat files. The DNA sequences displayed here represent dummy sequences and are identical to the sequences of the example test files co-supplied with the software.

The plant and fungal sciences community would benefit from a software tool that streamlines the generation of marker-specific Webin checklists, which is tedious to conduct by hand and difficult to code dynamically due to the idiosyncrasies of the different checklists (Fig 2). Specifically, it would be desirable to automate the conversion of EMBL- or GenBank-formatted flat files, which can be generated via various software suites for DNA sequence assembly and annotation (e.g., Artemis, DnaSP, Geneious), into correctly formatted Webin checklists that require nothing more but their upload to ENA via the interactive Webin submission system (Table 1). The software should hereby be available as an open-source and platform-independent tool that can be customized and expanded by other researchers. In the present investigation, we present such a tool. We report about the development and application of a Python package, entitled ‘EMBL2checklists’, that takes annotated DNA sequences of common plant and fungal DNA barcoding regions and associated metadata as input and returns properly-formatted checklists that are ready for data upload to ENA via the interactive Webin submission system.

**Table 1:** Overview of the bioinformatic steps involved in submitting novel DNA sequence data to ENA when using EMBL2checklists, starting from assembled DNA sequences.

	Bioinformatic step	Details of step	Manual processing	Available software
1	Sequence annotation	Sequence features and feature qualifiers using INSDC-compatible keywords are added to each DNA sequence.	high	Geneious^1^, Artemis^1^^,^^2^
2	Saving as flat file	Multiple annotated DNA sequences are saved as a single multi-sequence EMBL- or GenBank-formatted flat file.	minimal	Geneious^1^, Artemis^1^^,^^2^
3	Flat file validation	Flat file format, feature table syntax or taxonomic status of organism, among other aspects, are validated.	minimal	EMBL flat file validator^1^ (https://github.com/enasequence/sequencetools), SBOL validator^2^ (https://github.com/SynBioDex/SBOL-Validator)
4	Conversion to checklist	The EMBL- or GenBank-formatted flat file is converted into the Webin checklist file format.	minimal	EMBL2checklists^1^^,^^2^
5	Upload to ENA	The checklist is uploaded to ENA via the interactive route of the Webin submission system.	minimal	Any web browser

^1^For EMBL-formatted flat files;

^2^For GenBank-formatted flat files.

## Materials and methods

### Implemented checklist types

The software EMBL2checklists was designed to convert annotated DNA sequences and associated metadata into six different Webin checklist types. Sequence submission via the Webin submission system is primarily conducted through marker-specific checklists [15]. These checklists are pre-tailored to the idiosyncrasies of different genome regions, with different checklists displaying marker-specific customizations in order to capture the distinct information of the genomic regions under study (Fig 2). For example, a checklist that contains sequence information on the plastid *trnK/matK* region will be more complex than a checklist on the nuclear ribosomal external transcribed spacer (ETS) due to the location of the gene *matK* inside the group II intron of the tRNA gene for Lysine (*trnK*-UUU; [40]). A conversion of annotated DNA sequences into Webin checklists needs to take these marker-specific customizations into account. Thus, the Webin checklist for *trnK/matK* comprises eight mandatory columns, whereas the Webin checklist for the ETS comprises only five (Table 2). To enable submissions of a wide range of genomic regions to ENA, numerous marker-specific checklists have been implemented in Webin (https://www.ebi.ac.uk/ena/submit/annotation-checklists), but only a small number of these are relevant to plant and fungal DNA barcoding. The software EMBL2checklists is designed to convert annotated DNA sequences and associated metadata into one of six different Webin checklists. Given an EMBL- or GenBank-formatted flat file with the correct annotation features and feature qualifiers as input (Table 2), EMBL2checklists can generate marker-specific checklists for a series of DNA markers that are commonly employed in plant and fungal DNA barcoding (Fig 1). These markers are: (i) a common gene intron (e.g., *trnL* intron; [41]); (ii) a common intergenic spacer (IGS; e.g., *trnH-psbA*; [42]); (iii) the plastid *trnK/matK* region [43]; (iv) the nuclear ribosomal rRNA-encoding rDNA genes (i.e., 18S and 26S/28S rDNA; [36, 44]); (v) the nuclear ribosomal internal transcribed spacer (ITS; [28, 35]); and (vi) the nuclear ribosomal ETS ([45]). Coding regions that comprise a single exon (e.g., the plastid gene *rbcL* [42]) have not been implemented as separate checklists in EMBL2checklists due to the simplicity of their checklist layout; these checklists can be generated rapidly using a common spreadsheet editor and do not warrant an automated, software-driven construction. Plant biologists commonly use plastid and nuclear ribosomal DNA markers for DNA barcoding [26, 27], whereas fungal biologists almost exclusively rely on nuclear ribosomal DNA markers (especially the ITS and sections of the 28S rDNA) for DNA barcoding [35, 36]. Fungal DNA barcoding, thus, shares most of its barcoding markers with plant DNA barcoding, enabling the use of the same Webin checklist types and rendering EMBL2checklists relevant for plant and fungal biologists alike.

**Table 2:** Overview of the mandatory column name, annotation feature and feature qualifier specifications of the six checklist types implemented in EMBL2checklists.

Checklist name and description (Number of mandatory/optional columns)	Column name	Column value	Flat file feature	Feature qualifier
**gene_intron**—any gene intron (9/32)				
	GENE	str	gene	note
	5’_PARTIAL*	yes/no	intron	
	3’_PARTIAL*	yes/no	intron	
	5’_INTRON*	int	intron	
	3’_INTRON*	int	intron	
	NUMBER	int	intron	number
**IGS**—any intergenic spacer (7/33)				
	GENE1	str	misc_feature	product
	G1PRESENT	yes/no	gene	note
	GENE2	str	misc_feature	product
	G2PRESENT	yes/no	gene	note
**trnK_matK**—plastid *trnK/matK* gene region (8/23)				
	5’_CDS	yes/no	gene	note
	3’_CDS	yes/no	gene	note
	5’_PARTIAL	yes/no	gene	note
	3’_PARTIAL	yes/no	gene	note
	INTRON*	yes/no	intron, tRNA	gene
**rRNA**—18S/28S/5.8S nr rDNA gene (4/29)				
	SEDIMENT	18S/28S/5.8S	rRNA	product
**ITS**—nr internal transcribed spacer (7/24)				
	ISOLATION _SOURCE*	str	source	isolation _source
	18S	yes/no	rRNA	gene
	ITS1*	yes/no	misc_RNA	note
	ITS2*	yes/no	misc_RNA	note
	28S	yes/no	rRNA	gene
**ETS**—nr external transcribed spacer (5/28)				
	18S*	yes/no	rRNA	gene
	ETS_TYPE*	5’/3’	misc_RNA	note
	28S*	yes/no	rRNA	gene

Checklist and column names are displayed as defined by the Webin submission interface. The mandatory checklist columns ‘ORGANISM_NAME’, ‘ENV_SAMPLE’ and ‘SEQUENCE’ are present in all checklist types and, thus, are not displayed. The number of mandatory and optional checklist columns sums up to the total number of implemented columns for each checklist type. The application of special parsing rules (see main text) is indicated by an asterisk. For checklist type ‘rRNA’, any of the following sediment types are permitted: 5S, 5.8S, 12S, 16S, 18S, 23S, 25S, 26S, 28S. The column names and column values of each checklist are also presented in Fig 2, where they are cross-referenced with the structural characteristics of each DNA marker. Abbreviations used: nr = nuclear ribosomal; str = string; int = integer.

### Conversion of multiple sequence records

**Fig 3:**
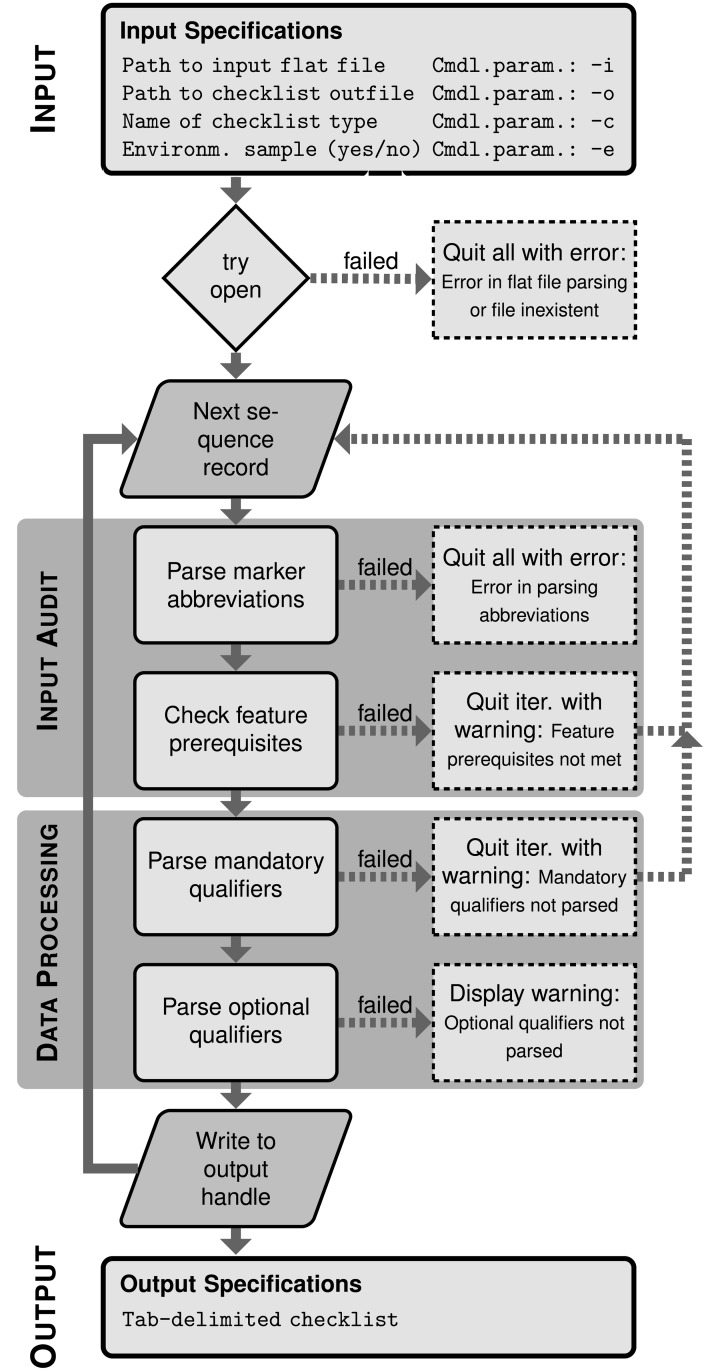
Overview of the internal structure of EMBL2checklists. The overview illustrates the two main processes that are executed sequentially for each sequence record: input audit and data processing. Rhomboid fields indicate the start and end points of the loop across sequence records. Fields with dotted outlines indicate states where standard data processing failed, which results either in aborting the current iteration (arrow back to next sequence record) or the software run as a whole. Abbreviations used: cmdl.param. = command-line parameter; iter. = iteration.

EMBL2checklists is able to convert multiple sequence records contained in an input flat file into a single Webin checklist. EMBL- or GenBank-formatted flat files may contain multiple sequence records, each with a specific set of annotation features and sequence metadata. EMBL2checklists accepts such a user-selected flat file as input, parses each sequence record individually, and writes the parsed information to the output file. Specifically, the software converts the sequence information contained in each sequence record into a separate row of the resulting checklist. Programmatically, EMBL2checklists parses the flat file via the BioPython library [46] and then iterates over the sequence records, processing one record at a time (Fig 3). During each iteration, the DNA sequence of a record, its annotation features, and its associated metadata are extracted and saved in form of a Python dictionary. Upon processing all sequence records, EMBL2checklists converts the dictionary of each successfully parsed record into a row of a pre-tailored, tab-delimited spreadsheet, the precise type of which had been selected by the user during software initialization. These rows are then appended to the output file collectively so that the number of rows written equals the number of sequence records successfully parsed from the input. This record-by-record processing of the input file allows the parsing algorithm to evaluate the sequence records individually and to skip specific records in the event of an error.

### Internal structure of software

EMBL2checklists conducts a series of data parsing steps that are executed sequentially for each sequence record and that include evaluations of feature prerequisites and the coherence between input data and checklist selection. The structure of EMBL2checklists consists of two main processes that are executed sequentially for each sequence record: input audit and data processing (Fig 3). Upon initialization, EMBL2checklists evaluates the presence, integrity, and syntax of the input flat file via the data parser of BioPython. Then, it proceeds to input audit and data processing. During input audit, two different checks are conducted on a given sequence record. These checks are implemented as separate functions and comprise the evaluation if (a) the DNA marker abbreviations found among the annotation features and their qualifiers are coherent with the user-selected checklist type (‘parse marker abbreviations’ in Fig 3), and (b) the sequence record contains the minimally necessary annotation features to generate a functional checklist (‘check feature prerequisites’). Thus, the input audit evaluates if a given sequence record contains the necessary annotation features, feature qualifiers and qualifier values for the chosen checklist type. For example, a sequence record of a ribosomal DNA region that does not contain at least one feature of class ‘rRNA’ in the feature table and in which that feature does not contain at least one qualifier of class ‘product’ is flagged as an error because information on the mandatory checklist qualifier ‘SEDIMENT’ cannot be parsed from such a record (Table 2). Sequence records that fail the evaluation of minimal feature prerequisites are skipped, whereas those that fail the parsing of correct marker abbreviations terminate the entire software execution (Fig 3) because the latter error is indicative of an incorrect checklist selection by the user. Upon successful input audit, data processing of the sequence record is started. During data processing, the annotation features and feature qualifiers of a sequence record relevant to the chosen checklist type are parsed. First, only those qualifiers are parsed that a valid checklist for the selected checklist type could not be generated without (i.e., ‘mandatory qualifiers’; Fig 3). Then, qualifiers are parsed, which are permissible but not required for the selected checklist type (i.e., ‘optional qualifiers’). During each parsing step, the spelling of feature and qualifier names is evaluated against an INSDC-compliant dictionary of feature definitions, which serves two purposes: first, it ensures that feature and qualifier names are spelled and formatted in compliance with the definitions of the INSDC [14]; second, it ensures the correct transfer of information from the annotation features into the columns of the resulting Webin checklists. If an error is encountered during data processing, the processing of that sequence record fails and raises an exception while communicating a short error message to the user. Upon data processing, the information of the current sequence record is saved into an output handle and the data audit for the next record initiated. Upon processing all sequence records, the writer function of EMBL2checklists appends the parsed information of each sequence record as independent rows to the output file.

### Special parsing rules

EMBL2checklists applies a series of special parsing rules in order to accommodate the idiosyncratic structure and information content of the different checklist types. Data processing is not homogeneous across all checklist types but includes the application of special parsing rules for select checklists. For the checklist type ‘gene_intron’, for example, the start and end position (‘5′_INTRON’ and ‘3′_INTRON’ in Table 2) and the completeness of the intron (‘5′_PARTIAL’ and ‘3′_PARTIAL’) is determined by the intron location information, not by its qualifier values. For checklist type ‘trnK_matK’, either an intron feature or a tRNA feature for gene *trnK*-UUU must be present in the sequence record. For checklist type ‘ITS’, two special rules apply: (a) if environmental samples are processed, the otherwise optional checklist column ‘ISOLATION_SOURCE’ becomes mandatory; and (b) the completeness of the rDNA gene 5.8S is inferred based on the presence of ITS1 and ITS2. For checklist type ‘ETS’, either a rRNA feature for the 18S and the 28S rDNA gene, or a misc_RNA feature with the info ‘5′ ETS’ or ‘3′ ETS’ must be present in the sequence record. To maintain an accurate implementation of these parsing rules across different software development stages, customary checks via the Python unit test framework [47] were added to the software.

### Input parameters and output specifications

**Fig 4:**
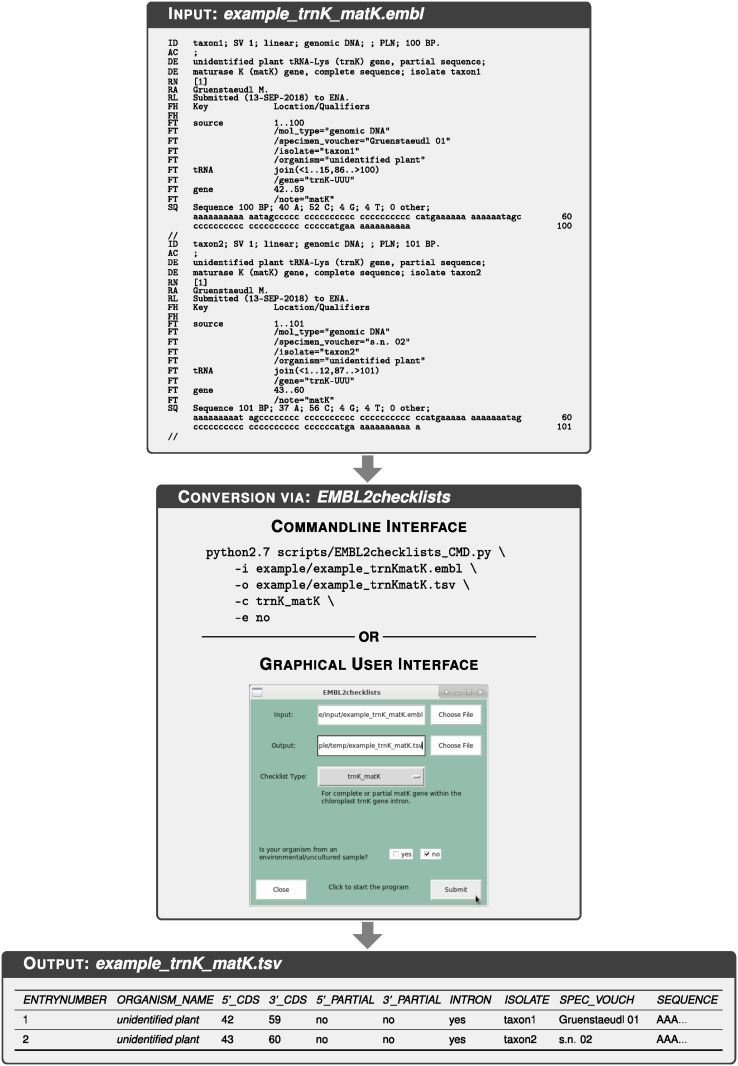
Overview of the conversion process from an EMBL-formatted flat file to a submission-ready Webin checklist via the application of EMBL2checklists. Name and content of the input and output files displayed are identical to the corresponding example test files co-supplied with the software.

Upon initializing the software, users of EMBL2checklists must specify four input parameters (Fig 4): (a) the name of the input flat file; (b) the name of the checklist output file; (c) the type of checklist selected by the user, and (d) a statement if the sequence records classify as environmental samples. Input parameter (a) must contain the name of, and the file path to, an EMBL- or GenBank-formatted flat file that comprises one or more sequence records. The precise file format of the input flat file is automatically identified based on the file ending (i.e., ‘.embl’ or ‘.gb’). The structure of each sequence record must be compliant with the identified flat file format and consist of a multi-line feature table, followed by the interleaved DNA sequence. The source feature, which pertains to the sequence as a whole and often contains sequence metadata, must be located at the top of the feature table. Annotation features, which indicate the type and boundaries of localized sequence features, must be located below the source feature. Input parameter (b) must contain the name of, and the file path to, the output checklist. The checklists generated by EMBL2checklists are human-readable and can be edited by any common spreadsheet editor such as Microsoft Excel (Microsoft Corporation, Redmond, WA, USA) or LibreOffice (The Document Foundation, Berlin, Germany). If all sequence records of the input file are processed correctly, the output checklist displays as many rows as the number of sequence records in the input file (disregarding the title row of the checklist). Input parameter (c) must be the name of one of six checklist types implemented in EMBL2checklists and specifies the rule set applied during input audit and data parsing of the sequence records. Input parameter (d) must be either ‘yes’ or ‘no’ and specifies the classification of all sequence records of an input file as environmental samples. This parameter is implemented in EMBL2checklists because its information is mandatory for certain Webin checklist types. It is typically answered with ‘yes’ if the DNA sequences under study were generated as part of a metabarcoding experiment. Upon specifying each of these parameters, EMBL2checklists begins to process the input file.

### Commandline and graphical user interface

EMBL2checklists was developed for classical biologists and bioinformaticians alike. Thus, the software is equipped with a graphical user interface (GUI) as well as a command-line interface (CLI) for its operation (Fig 4). The GUI is based on the Python library Tkinter (http://infohost.nmt.edu/tcc/help/pubs/tkinter/web/index.html) and designed to provide an intuitive and easy-to-use interface that allows users with little or no bioinformatics knowledge to operate the software. Due to the use of Tkinter, the GUI of EMBL2checklists is virtually identical under Windows, MacOS, and Linux operating systems (S2 Fig), enabling a consistent GUI performance across the three platforms. To execute EMBL2checklists via the GUI, users enter the four input parameters via the available input fields and drop-down menus. To receive help and detailed explanations via the GUI, users may hover their mouse pointer over a field of interest, which initiates a help bubble next to the pointer. Moreover, details of individual checklist types are automatically displayed upon selecting one of the implemented checklists from the drop-down menu. If EMBL2checklists raises an exception while operating under the GUI, an error message is printed to a pop-up window of the GUI. The GUI can be accessed via file ‘EMBL2checklists_GUI.py’ of the scripts folder or via the command ‘EMBL2checklists_GUI’ upon proper package installation. More information on the design and functionality of the GUI of EMBL2checklists is available in [48]. The CLI employs functions of the Python library argparse (https://pypi.org/project/argparse/) and allows more experienced users to execute the software via the command-line and to integrate the software into larger bioinformatic workflows. To execute EMBL2checklists via the CLI, users specify the name of the input flat file via command-line argument ‘-i’, the name of the checklist output file via argument ‘-o’, the type of checklist via argument ‘-c’, and the classification of the sequence records as environmental samples via argument ‘-e’. To receive help and detailed explanations, CLI users may invoke command-line argument ‘-h’ (i.e., ‘EMBL2checklists_CLI -h’). If EMBL2checklists raises an exception while operating under the CLI, an error message is printed to the standard output stream. The CLI can be accessed via file ‘EMBL2checklists_CLI.py’ of the scripts folder or via the command ‘EMBL2checklists_CLI’ upon proper package installation.

### Development, installation and compatibility

EMBL2checklists was written in Python 2.7 [49] and is, thus, platform independent. It can be executed on any system equipped with a Python 2 compiler and upon the installation of the necessary Python dependencies. The software uses three separate Python packages as dependencies: Biopython, argparse and Tkinter. EMBL2checklists is open source and released under the BSD 3-Clause license (https://opensource.org/licenses/BSD-3-Clause). Other bioinformaticians are, thus, allowed to expand and customize the software to fit their own data submission needs, including the development of functions to parse additional checklist types. EMBL2checklists is available via the Python Package Index (https://pypi.org/project/EMBL2checklists/) and can be installed via any PyPI-compatible package management system for Python 2 such as pip (https://pip.pypa.io) or setuptools (https://pypi.org/project/setuptools/). For example, users may type the following command in a terminal to install EMBL2checklists on their system:

$ pip2 install EMBL2checklists

During installation via pip or setuptools, the setup script generates executables for both CLI and GUI initialization and places these into the Python search path for scripts and modules (i.e., the PYTHONPATH). EMBL2checklists was successfully tested on a Windows (Microsoft Windows 10), a MacOS (MacOS 10.14—Mojave) and three different Linux environments (Arch Linux 4.18, Debian 9.0 and Ubuntu 18.10). Moreover, the compatibility of EMBL2checklists to the Python interpreters of different operating systems was confirmed through continuous integration for Windows via Appveyor (https://ci.appveyor.com/projects) and for MacOS and Linux via Travis-CI (https://travis-ci.com/michaelgruenstaeudl/). In addition, code stability across different versions of this software was tested and maintained through the application of Python unit tests [47].

### Usage

For a typical execution of EMBL2checklists via the CLI, a user may type the following command in a terminal:

$ EMBL2checklists_CLI

 -i example/example_trnKmatK.embl \

 -o example/example_trnKmatK.tsv \

 -c trnK_matK \

 -e no

For a typical execution of EMBL2checklists via the GUI, a user may select the input and output file, the checklist type and the classification of all sequences as environmental samples manually upon initializing the GUI, which is achieved by typing the following command in a terminal:

$ EMBL2checklists_GUI

Windows users may further initialize the GUI by double-clicking the executable file ‘EMBL2checklists_GUI.exe’, which is built upon package installation. A step-by-step protocol of the bioinformatic steps necessary to generate submission-ready checklist files is provided on protocols.io (http://dx.doi.org/10.17504/protocols.io.v6me9c6). The protocol provides instructions on installation and data processing, links to recommended software tools and video tutorials, examples of input and output files, and animations of CLI and GUI usage.

## Results and discussion

### Application of software on empirical data

The utility of EMBL2checklists to plant and fungal biology is illustrated by its application in the submission process of DNA sequences to ENA by four recent investigations. Specifically, EMBL2checklists was employed for the submission preparation of several hundred DNA sequences in three plant phylogenetic [50–52] and one fungal metagenomic investigation [53]. The plant phylogenetic investigations utilized common plant DNA barcoding markers to infer the phylogenetic history of select plant lineages; the fungal metagenomic investigation utilized nuclear ribosomal DNA barcodes to characterize arbuscular mycorrhizal soil fungi. In each case, EMBL2checklists was used to convert flat files in GenBank format that were generated from sets of assembled and annotated sequences via the software suite Geneious. Upon conversion to checklists, the sequence data was uploaded to ENA via the interactive Webin submission system, and accession numbers were received from ENA by email within less than 48 hours of submission. Thus, preparing and submitting the sequence dataset of an investigation via EMBL2checklists can be conducted within the typical time frame of a minor manuscript revision.

### Post-processing of checklists and work-sharing

Due to the data format of Webin checklists and the structure of the interactive Webin submission process, the use of EMBL2checklists for sequence submissions to ENA displays two special advantages: it allows the post-processing of checklists, and it enables work-sharing during the submission process. First, the data format of Webin checklists is human-readable and, thus, allows the manual addition of column information, should users wish to augment the checklist prior to submission. Specifically, the easily-accessible checklist data structure allows users to modify or append column content, as long as the name and order of existing columns remain unchanged. For example, users who wish to add information about the geographic location of one or more sequences after having processed the sequence data with EMBL2checklists can simply add a column entitled ‘LOCALITY’ before column ‘SEQUENCE’ of the checklist and add geographic location information for one or more sequences. Likewise, users who wish to combine multiple checklists can do so, as long as the number and order of columns are identical (and the title row of the second checklist is removed). Second, EMBL2checklists allows the implementation of a work-sharing strategy during the preparation of sequence submissions because the information contained within the identification, description and reference lines of a sequence record (such as author name or author institution) is not saved as part of the checklist output. The software only processes the feature table and the DNA sequence of a sequence record. Ancillary information of a dataset such as author name or study title must be associated with the sequence data during the interactive submission process. Specifically, personal and institutional information of the submitter is associated with the data through the Webin submission service prior to data upload, irrespective of the checklist type. Hence, EMBL2checklists does not need to be executed by the same person that conducts the data upload or has generated the sequence but allows a work-sharing strategy in which one person (or section of a workflow) conducts the data conversion via EMBL2checklists, while another person (or section of a workflow) conducts the data submission. Work-sharing may be helpful if the sequence submission process is centralized within a lab or academic institution, allowing those researchers that prepare the data for submission to ENA to be different from those that actually conduct the data upload.

### Data converters and other ENA submission strategies

The paucity of file formats acceptable for data submission to public sequence databases is one of the main bottlenecks in the effort to increase participation in public data sharing and has spurred the recent development of various data converters. The software EMBL2checklists is one of several current projects that aim to provide automated data conversion between the EMBL or GenBank flat file format and data formats that are commonly parsed by biological software and databases [21, 54, 55]. The underlying aim of many of these projects is to simplify the conversion process of sequence data into file formats that are accepted during submission to public sequence databases [21, 54]. Given the custom validation criteria and the idiosyncratic submission procedures employed by many of these databases, such data converters represent an important means to enable user-friendly data submissions, at least until such time as submission procedures across INSDC databases are standardized [56]. EMBL2checklists was specifically designed to bridge the gap between common software suites for DNA sequence assembly and annotation (e.g., Artemis, DnaSP, Geneious) and the interactive Webin submission process. Compared to most other recent data converters, EMBL2checklists supports the conversion of flat files that contain multiple sequence records. At the same time, EMBL2checklists aims to fulfill a seemingly counterintuitive task: The software converts EMBL- or GenBank-formatted flat files into Webin checklists, which the receiving database of ENA eventually converts back into the flat file format. Theoretically, it would be easier to upload annotated DNA sequences in EMBL flat file format to ENA directly, which can be accomplished via the programmatic and command-line submission services of ENA [12, 25]. In practice, however, both of these submission routes exclude a considerable number of regular users due to the bioinformatics expertise required. Many ordinary users, thus, tend to submit their DNA sequences to repositories with a more user-friendly toolkit for submission preparation (e.g., GenBank). Given that the databases of GenBank, ENA and DDBJ are synchronized daily to ensure ubiquitous data accessibility [14], the preferential submission of sequences to GenBank on the grounds of submission convenience does not affect data availability. A more even data submission across the three INSDC databases may, however, be desirable on the grounds of load balancing [57], resource mirroring [58], and equal turnaround times for helpdesk requests [25]. Until methods are in place that allow a more user-friendly upload of annotated DNA sequences in flat file format to ENA, EMBL2checklists represents one of the most intuitive options for plant and fungal biologists without bioinformatics experience to automatically generate submission-ready checklists.

## Conclusion

The lack in automated conversion between EMBL- or GenBank formatted flat files and submission-ready Webin checklists represented a gap that compelled many researchers to conduct manual data processing before submitting data to the public sequence database ENA. By developing the software EMBL2checklists, we have filled this gap. EMBL2checklists is designed as an easy-to-use software application that bridges the gap between common software suites for DNA sequence assembly and annotation and the interactive data submission process of ENA. The software converts annotated DNA sequences plus associated metadata into properly-formatted Webin checklists. Specifically, the software takes the idiosyncrasies of marker-specific checklist types into account and generates submission-ready checklists for specifically those DNA markers that are commonly employed in plant and fungal DNA barcoding. Thus, EMBL2checklists can be employed to prepare the most common plant and fungal DNA barcoding marker sequences for upload and submission to ENA via the interactive Webin submission system. Users may generate input files for our software through any of several common sequence analysis environments (e.g., Artemis, DnaSP, Geneious) and then employ the GUI of EMBL2checklists to prepare their sequence submissions. Upon processing with EMBL2checklists, the user receives a checklist that can be directly uploaded to ENA or further edited with a common spreadsheet editor. The utility of EMBL2checklists is best illustrated by its application during the submission process of hundreds of DNA sequences to ENA in four recent investigations [50–53]. With the development of EMBL2checklists, we hope to provide a useful software tool to biologists and bioinformaticians alike, increase the amount of sequence data deposited to public sequence databases [7] and advance the idea of publicly-shared research data [9, 10]. By extension, we believe that EMBL2checklists may play an important role in future data management and data stewardship of plant and fungal DNA sequence data under the FAIR data principle [59, 60].

## Supporting information

S1 Fig The number of DNA sequences of ENA’s fungal taxonomic division (FUN) of the standard annotated assembled sequence data class (STD) released on ENA per calendar year, displayed by type of DNA barcoding marker. Due to the daily synchronization of sequence records between GenBank, ENA, and DDBJ, the release numbers are identical across all three databases.
(PDF)

S2 Fig The appearance of the GUI of EMBL2checklists under different operating systems. The respective operating system is listed below each image.
(PDF)
