# Supplementary material for: EMBL2checklists: A Python package to facilitate the user-friendly submission of plant and fungal DNA barcoding sequences to ENA
Source: PLoS One. 2019 Jan 10;14(1):e0210347. doi: 10.1371/journal.pone.0210347 (PMC6328100; doi:10.1371/journal.pone.0210347)

Supplemental Material

EMBL2checklists: A Python package to facilitate the user-friendly submission of plant and fungal DNA barcoding sequences to ENA

Michael Gruenstaeudl[1*], Yannick Hartmaring[2]
[1] Institut für Biologie, Freie Universität Berlin, 14195 Berlin, Germany
[2] Institut für Bioinformatik, Freie Universität Berlin, 14195 Berlin, Germany
* m.gruenstaeudl@fu-berlin.de

Supplemental Figure 1. Number of DNA sequences of ENA's fungal taxonomic division (FUN) of the standard annotated assembled sequence data class (STD) released on ENA per calendar year, displayed by type of DNA barcoding marker. Due to the daily synchronization of sequence records between GenBank, ENA and DDBJ, the release numbers are identical across all three databases.

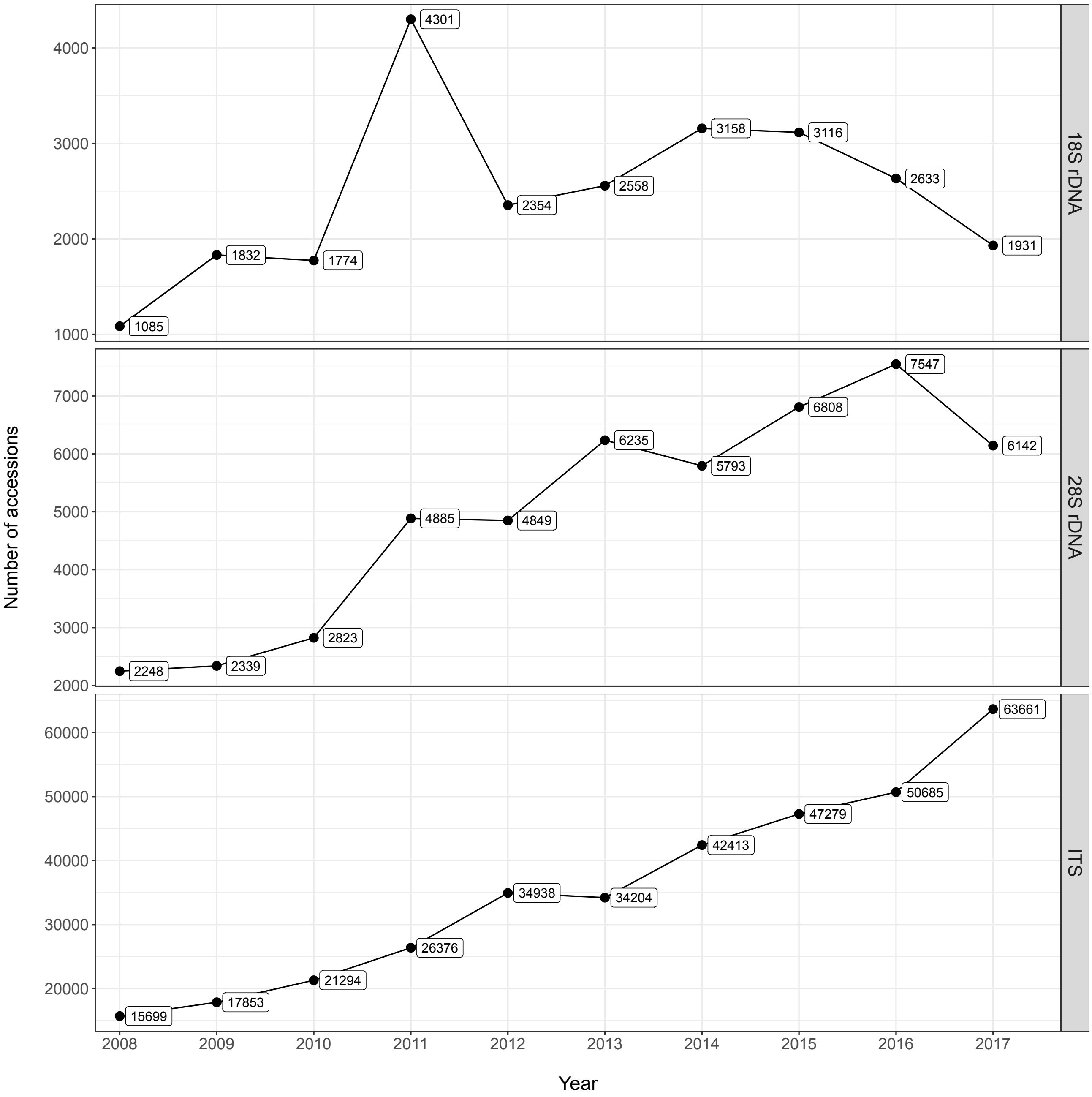

Supplement: S1 Fig — Due to the daily synchronization of sequence records between GenBank, ENA, and DDBJ, the release numbers are identical across all three databases. (PDF) [file pone.0210347.s001.pdf]
