# Supplementary material for: EMBL2checklists: A Python package to facilitate the user-friendly submission of plant and fungal DNA barcoding sequences to ENA
Source: PLoS One. 2019 Jan 10;14(1):e0210347. doi: 10.1371/journal.pone.0210347 (PMC6328100; doi:10.1371/journal.pone.0210347)
Supplement: S2 Fig — The respective operating system is listed below each image. (PDF) [file pone.0210347.s002.pdf]

SUPPLEMENTAL MATERIAL

EMBL2checklists: A Python package to facilitate the user-friendly submission of plant and fungal DNA barcoding sequences to ENA

Michael Gruenstaeudl[1*], Yannick Hartmaring[2]
[1] Institut für Biologie, Freie Universität Berlin, 14195 Berlin, Germany
[2] Institut für Bioinformatik, Freie Universität Berlin, 14195 Berlin, Germany
* m.gruenstaeudl@fu-berlin.de

Supplemental Figure 2. Appearance of the GUI of EMBL2checklists under different operating systems. The respective operating system is listed below each image.

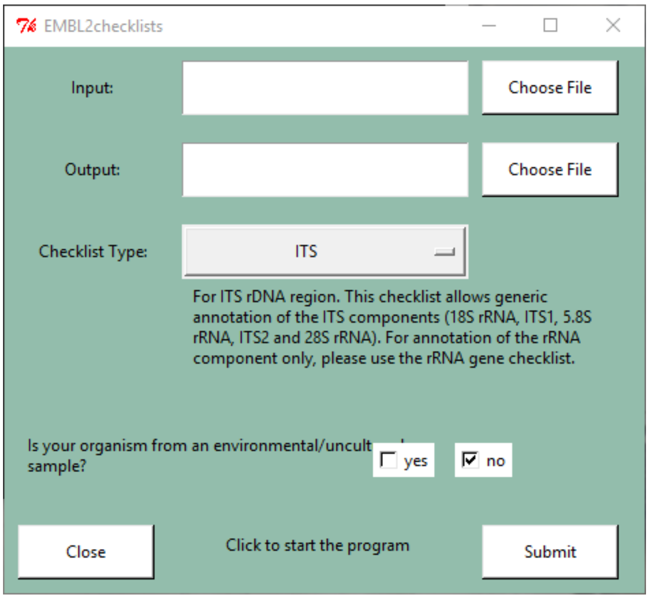

Windows 10

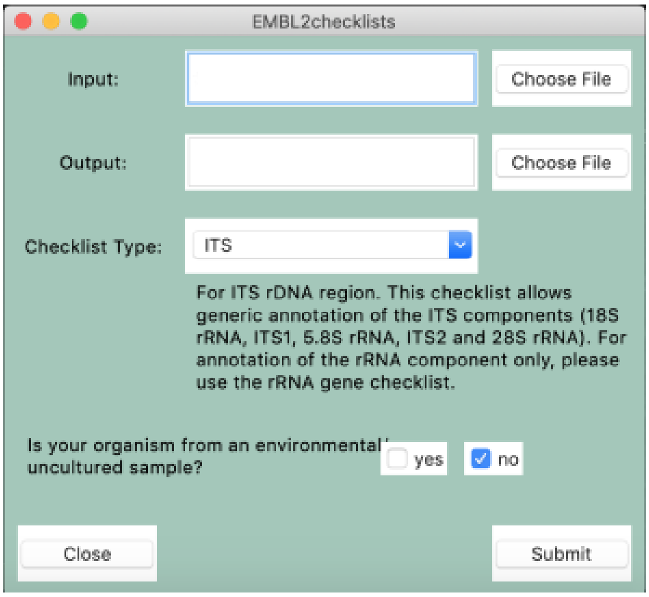

MacOS Mojave

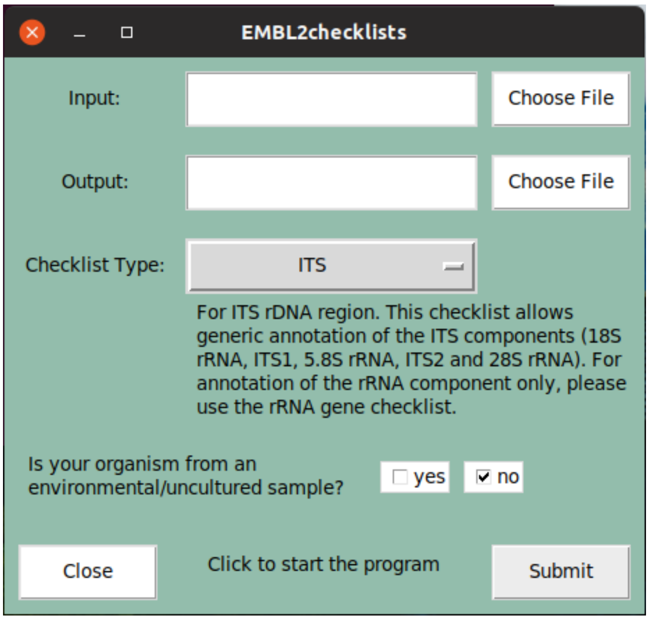

Ubuntu 18.10

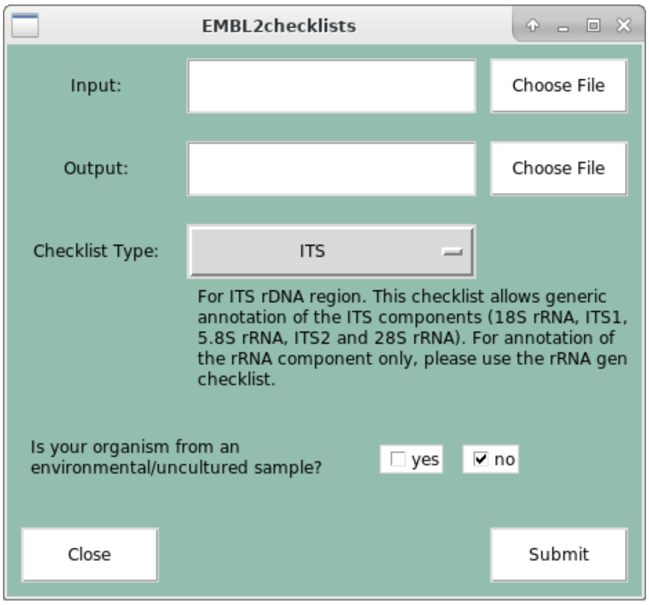

ArchLinux 4.19
